# Presentation and management of pulmonary artery sarcoma

**DOI:** 10.1186/s13569-014-0019-2

**Published:** 2015-01-21

**Authors:** Han Hsi Wong, Ioannis Gounaris, Ann McCormack, Marius Berman, Dochka Davidson, Gail Horan, Joanna Pepke-Zaba, David Jenkins, Helena M Earl, Helen M Hatcher

**Affiliations:** Addenbrooke’s Hospital, Cambridge University Hospitals NHS Foundation Trust, Box 193, Cambridge Biomedical Campus, Hills Road, Cambridge, CB2 0QQ UK; Papworth Hospital NHS Foundation Trust, Papworth Everard, Cambridge, CB23 3RE UK; University of Cambridge Department of Oncology and NIHR Cambridge Biomedical Research Centre, Hills Road, Cambridge, CB2 0QQ UK

**Keywords:** Pulmonary artery sarcoma, Intimal sarcoma, Signs, Symptoms, Pulmonary endarterectomy, Treatment, Survival

## Abstract

**Background:**

Pulmonary artery sarcoma (PAS) is a rare but aggressive malignancy that leads to heart failure and death without treatment. Here we reviewed the presentation and management of patients treated at a national centre for pulmonary endarterectomy (PEA) and its associated hospital in Cambridge, UK.

**Methods:**

Details of PAS patients treated at Papworth and Addenbrooke’s Hospitals between 2000 and 2014 were reviewed.

**Results:**

Twenty patients were diagnosed with PAS (11 males, 9 females), with a median age of presentation of 57 years (range 27–77). Presenting symptoms include dyspnoea (20), chest pain/tightness (7), oedema (5), constitutional symptoms (5), cough (3) and haemoptysis (3). Twelve patients were in group III/IV of the NYHA functional classification of symptoms. Initial CT scans were suggestive of thromboembolism in seven patients. Histological findings were of intimal sarcoma (13) and high grade sarcoma NOS (6).

Median overall survival (OS) was 17 months. Fourteen patients underwent PEA to relieve vascular obstruction, while six had inoperable and/or metastatic disease. There were three peri-operative deaths. Although there was no difference in median OS between patients who had PEA and those who did not (20 vs 17 months, P = 0.2488), surgery provided significant symptomatic improvement and some with long-term survival. Five patients received post-surgical chemotherapy (anthracycline +/− ifosfamide), and after completion four also had radiotherapy. Patients who received post-operative chemo- and radio-therapy showed a trend towards better survival compared to those who had surgery alone (24 vs 8 months, P = 0.3417). For palliative chemotherapy, partial responses were observed with the VID regimen and pegylated liposomal doxorubicin. Stable disease was achieved in a patient with intimal sarcoma with rhabdomyosarcomatous differentiation on third-line cisplatin and topotecan. The longest surviving patient (102 months) has had PEA, adjuvant epirubicin and radiotherapy. She developed lung metastases 7 years later, which were treated with radiofrequency ablation.

**Conclusions:**

PAS often presents with symptoms mimicking pulmonary hypertension, heart failure or thromboembolic disease. PEA provides good symptomatic relief and in some cases, offers a chance of long-term survival. Although outcome appears to be better when PEA is combined with post-operative chemo- and radio-therapy, further studies are warranted.

## Background

First described by Moritz Mandelstamm from an autopsy in 1923, primary pulmonary artery sarcoma (PAS) is an uncommon but increasingly recognised thoracic malignancy, with only a few hundred cases reported in the literature [1-3]. The aetiology is unknown and the disease results in significant morbidity and high mortality. The clinical and radiological findings are often similar to those of thromboembolic disease, leading to delays in confirming the diagnosis. Typically the disease presents in adulthood with symptoms including dyspnoea, cough, haemoptysis, chest pain and weight loss.

Given the rarity of PAS only case reports and small case series have been published, the majority focusing on histopathological appearances and surgical aspects of its management [2-12]. Surgery remains the mainstay of management for patients with PAS, and can include pulmonary endarterectomy (PEA), lobectomy and pneumonectomy [2,3,6]. The role of additional chemotherapy and radiotherapy after surgical resection remains largely unproven.

In this study, we report our experience in managing 20 patients with PAS over a period of 14 years at Papworth Hospital, a national centre for PEA and its associated Oncology Department at Addenbrooke’s Hospital in Cambridge, UK. The 20 patients described in this report constitute one of the largest series in the published literature.

## Methods

### Patient groups

Papworth Hospital in Cambridge, UK is a specialist cardiothoracic centre. It is associated with Addenbrooke’s Hospital which provides radiotherapy and systemic therapy to the regional sarcoma population. Patients with PAS treated at these two hospitals between the years of 2000 and 2014 were identified retrospectively. Diagnosis was confirmed by histological examination and immunohistochemical staining. Patients’ clinical records were obtained and examined. All 20 patients identified were included in the study.

### Statistical analysis

Survival analysis was performed using the Kaplan-Meier method and log-rank test. Surviving patients were censored at last contact. A P value of <0.05 was considered to be statistically significant.

## Results

### Patient characteristics

**Figure 1:**
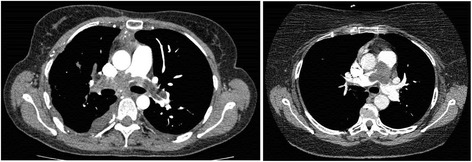
**Pulmonary artery intimal sarcoma mimicking pulmonary embolism.** CT images of two patients who presented with dyspnoea and dependent oedema.

A total of 20 patients were treated at our centres between 2000 and 2014. The patient characteristics are summarised in Table 1. Eleven (55%) patients were male and the age at presentation ranged from 27 to 77 years, with a median of 57 years. The vast majority of patients presented with symptoms consistent with acute or chronic pulmonary hypertension such as dyspnoea (New York Heart Association (NYHA) functional class III and IV in 60% of cases) and chest pain. Some experienced constitutional symptoms of fever, night sweats, anorexia and weight loss. Intimal sarcoma was the histological diagnosis for the majority of the patients (65%), while high-grade sarcoma NOS was found in 35%. We were unable to obtain tumour tissue from an elderly patient with significant co-morbidities despite radiologically-guided biopsy, although his CT scan was unequivocal of PAS. In seven (35%) of the total cases, initial CT images were suggestive of pulmonary emboli (Figure 1).

**Table 1:** **Patient characteristics (n = 20)**

**Variables**	**No. of patients (%)**
**Gender**	
- Male	11 (55%)
- Female	9 (45%)
**Median age (years)**	57 (range 27 – 77)
- Male	64 (range 27 – 77)
- Female	57 (range 27 – 70)
**Presenting symptoms**	
- Dyspnoea	20 (100%)
- Chest pain/tightness	7 (35%)
- Dependent oedema	5 (25%)
- Constitutional symptoms	5 (25%)
- Cough	3 (15%)
- Haemoptysis	3 (15%)
**New York Heart Association functional classification**	
- I	0
- II	8 (40%)
- III	8 (40%)
- IV	4 (20%)
**Clinical signs suggestive of pulmonary hypertension**	
- Present	16 (80%)
- Absent	4 (20%)
**Pulmonary artery involvement**	
- Unilateral	3 (15%)
- Bilateral	17 (85%)
**Disease stage**	
- Operable and non-metastatic	14 (70%)
- Inoperable and/or metastatic	6 (30%)
**Histological subtype**	
- Intimal sarcoma	13 (65%)
- High-grade sarcoma NOS	6 (30%)
- Unable to obtain tissue for diagnosis	1 (5%)

### Treatments

**Figure 2:**
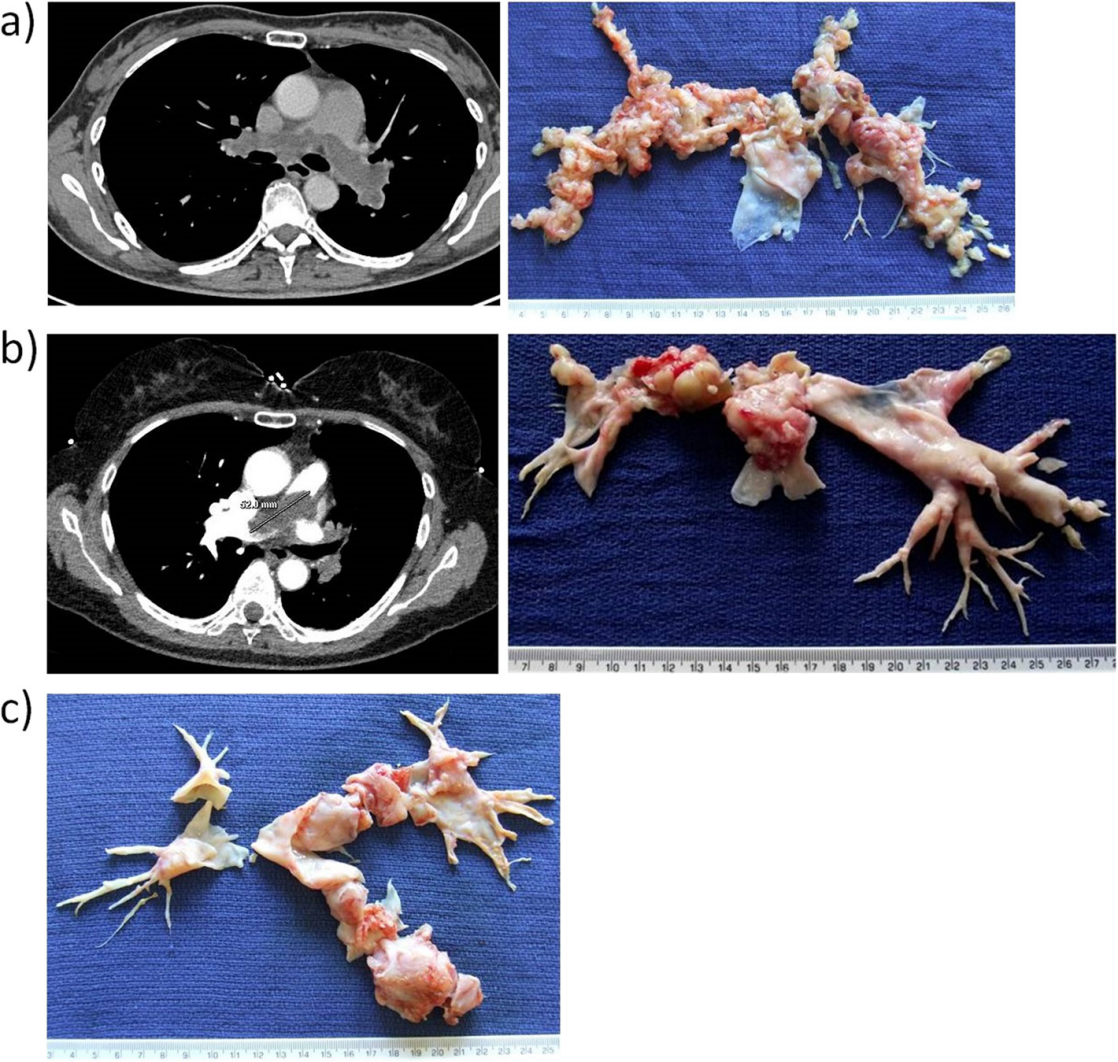
**Pulmonary endarterectomy for pulmonary artery sarcoma. (a)** CT image and resected specimen of a diffuse, bilateral pulmonary artery sarcoma. **(b)** CT image and resected specimen of a saddle-type pulmonary artery sarcoma. **(c)** Resected specimen of a left-sided, intraluminal pulmonary artery sarcoma.

Table 2 summarises the treatments given. Fourteen (70%) patients underwent PEA, while the remaining six (30%) had inoperable (secondary to mediastinal invasion or extension into lung parenchyma) and/or metastatic disease. PEA (Figure 2) is a high-risk procedure involving cardiopulmonary bypass and circulatory arrest under deep hypothermia (18–20°C). There were three peri-operative deaths (two of which had emergency surgery due to rapid cardiovascular and respiratory deterioration but died on the same day). However, the 11 patients who underwent and survived PEA reported significant improvements in their symptoms (all had post-operative NYHA score of I/II). Immediate post-operative morbidities included atrial fibrillation, chest infection, headache of uncertain aetiology, and haemothorax that resolved after drainage. The median length of hospital stay was 18 days (range 10 to 21 days). All resections were of R1 or R2.

**Table 2:** **Treatment modality (n = 20)**

**Variables**	**No. of patients**
**Operable and non-metastatic disease**	
- Pulmonary endarterectomy	14
- Post-operative chemotherapy	5
- Post-operative radiotherapy	4
**Inoperable, metastatic or recurrent disease**	
- Chemotherapy	5
- Radiotherapy	5
- Radiofrequency ablation	1

Five patients received post-operative chemotherapy (Table 3) – four of them had small volume metastases and the other had no macroscopic residual disease. The agents used were epirubicin, doxorubicin and/or ifosfamide. Three patients received post-operative anthracyclines which they tolerated without clinical cardiotoxicity. After chemotherapy, four patients also received immediate radiotherapy – of these, three patients had metastatic lung nodules and were given palliative doses of 30–36 Gy using parallel opposed fields to the primary sites for local control. The remaining patient, who showed no evidence of disease after PEA and adjuvant epirubicin, was given a radical dose of 60 Gy in 30 fractions by three-dimensional conformal radiotherapy.

**Table 3:** **Chemotherapy regimens and response**

**Chemotherapy**	**No. of patients**	**Best radiological response**
**Post-operative**		
- Epirubicin	1	No evidence of disease
	1	Stable disease
- Doxorubicin	1	Progressive disease
- Ifosfamide	1	Stable disease
- Doxorubicin + ifosfamide	1	Stable disease
**Palliative (first-line for inoperable and/or metastatic disease)**		
- Ifosfamide	1	Progressive disease
- Pegylated liposomal doxorubicin hydrochloride (PLDH)	1	Partial response
- Vincristine + ifosfamide + doxorubicin (VID)	1	Partial response
- Paclitaxel	1	Not evaluable
**Palliative (second- and subsequent-line)**		
- Cisplatin + topotecan	1	Stable disease
- Epirubicin	1	Progressive disease
- Ifosfamide	1	Progressive disease
- Ifosfamide + etoposide	1	Progressive disease
- Paclitaxel	1	Progressive disease

**Figure 3:**
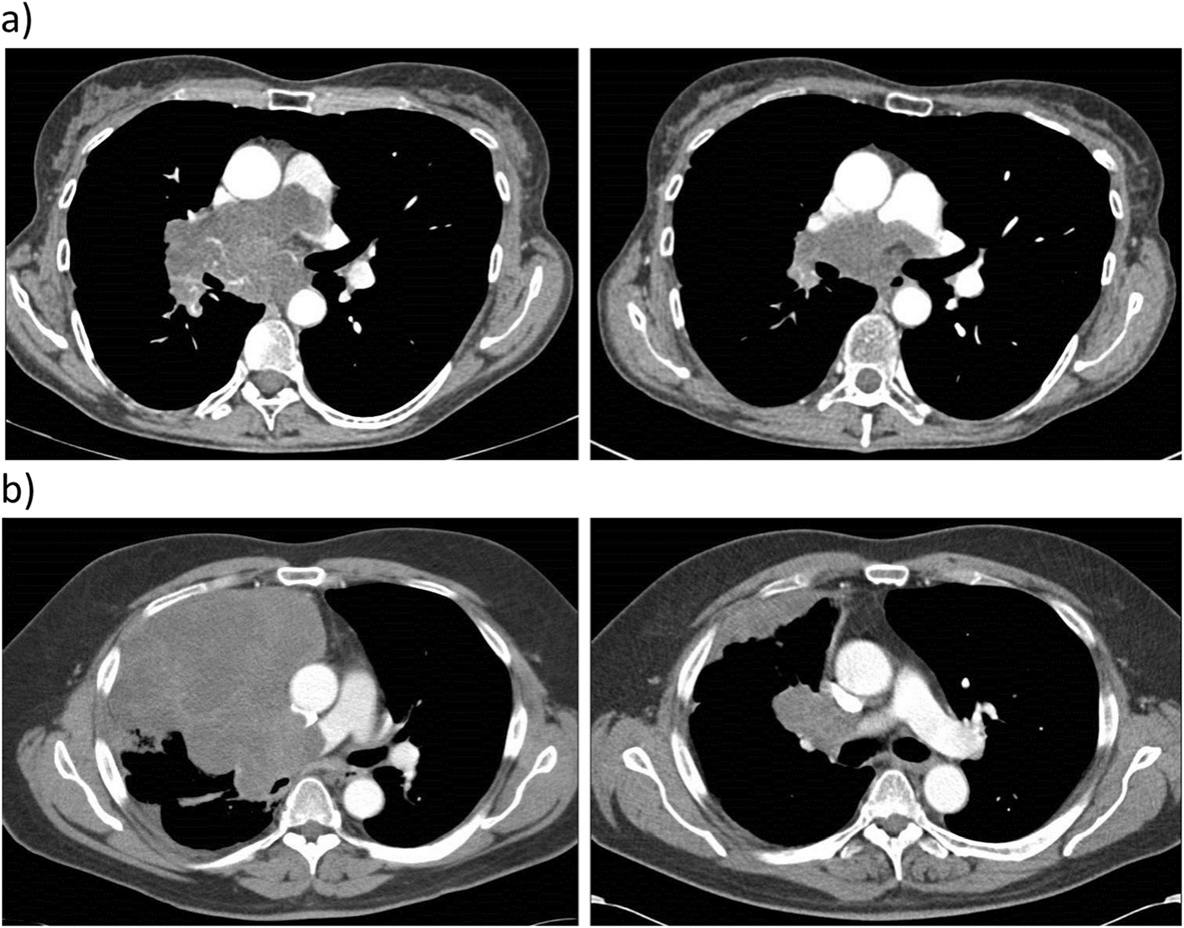
**CT images of response to first-line palliative chemotherapy for inoperable and metastatic pulmonary artery sarcoma. (a)** A 57-year-old woman with intimal sarcoma after three cycles of pegylated liposomal doxorubicin hydrochloride. **(b)** A 64-year-old man with intimal sarcoma with rhabdomyosarcomatous differentiation after six cycles of vincristine, ifosfamide and doxorubicin chemotherapy.

Patients with inoperable and/or metastatic disease showed benefit from radiotherapy for locoregional control – four patients had disease stabilisation and one demonstrated tumour shrinkage when it was combined with chemotherapy. For those patients treated with palliative chemotherapy, partial responses were observed with vincristine, ifosfamide and doxorubicin (VID), and pegylated liposomal doxorubicin hydrochloride (PLDH) (Figure 3), although the time to progression was only 6 and 5 months, respectively. In two patients anthracycline-based chemotherapy could not be given as a first-line treatment secondary to cardiac dysfunction; one of the two received single-agent paclitaxel but had to stop treatment due to significant neutropaenia after only one dose (Table 3). Stable disease that lasted 3 months was achieved in a patient with intimal sarcoma with rhabdomyosarcomatous differentiation using third-line cisplatin and topotecan.

Of the 20 patients with PAS, six received post-operative or palliative therapy at our centre. The remaining 11 patients (after excluding three peri-operative deaths) received their oncological treatments back at their referring hospitals. Although we were able to access the majority of the data, we could not obtain the complete information for six patients – one patient was discharged back to her local hospital after PEA but her post-operative treatment is unknown; one patient with inoperable and metastatic disease was transferred back to her referring hospital for palliative treatment but the details are lacking; two patients received palliative radiotherapy for recurrence but we cannot be certain whether they also had chemotherapy; and we do not have the chemo- and radio-therapy details of two patients who were treated for disease relapse.

### Survival

**Figure 4:**
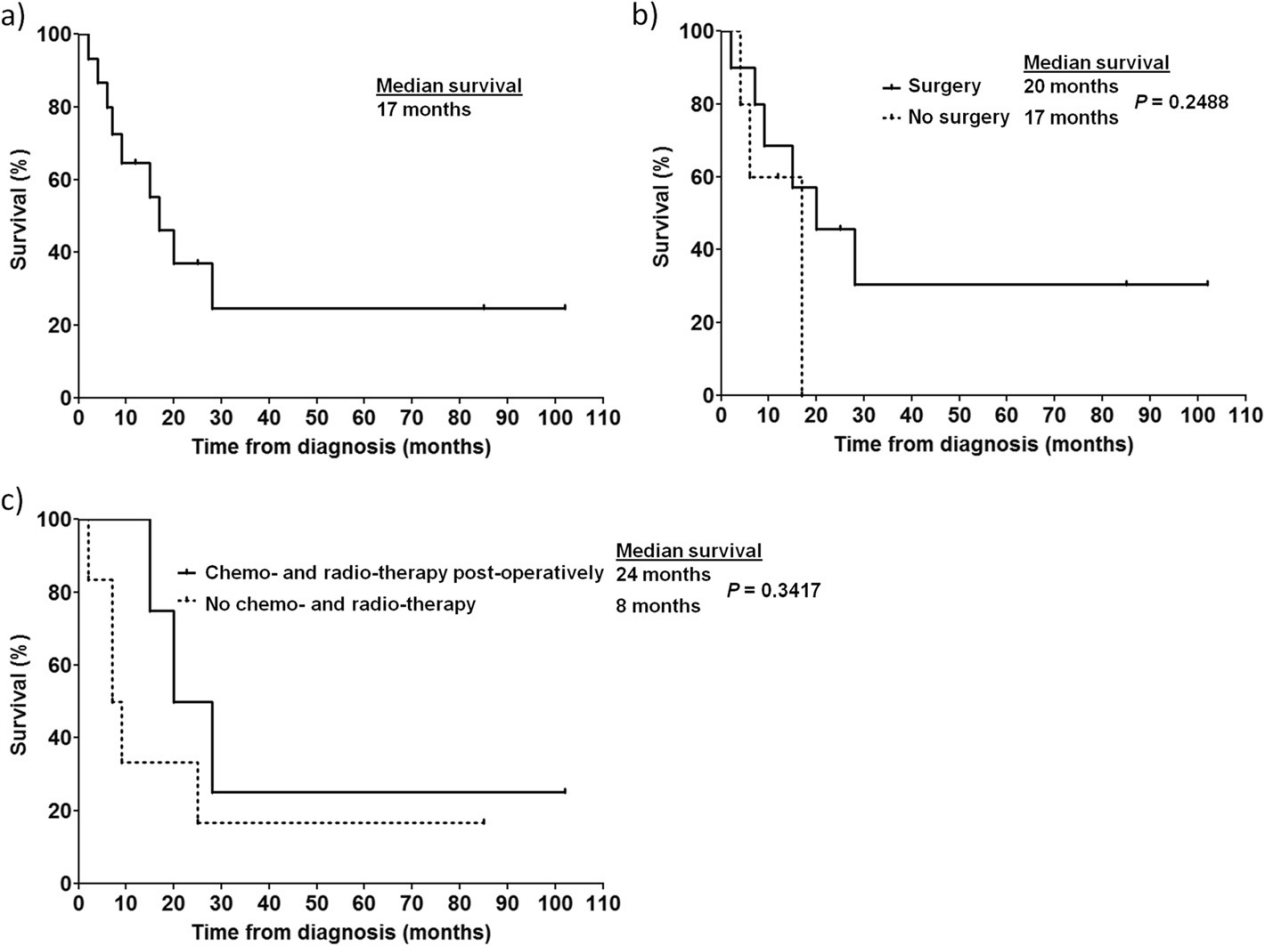
**Overall survival of patients with pulmonary artery sarcoma.** Kaplan-Meier plots for: **(a)** all patients in the series (n = 20), **(b)** patients who underwent PEA (n = 14) compared to those who did not (n = 6), and **(c)** patients who received both post-operative chemo- and radio-therapy (n = 4) compared to those who did not (n = 7 – six had surgery only and one had post-operative chemotherapy alone. Three patients who died peri-operatively were excluded).

The median overall survival (OS) for all 20 patients was 17 months (Figure 4). There was no difference in median OS between patients who underwent PEA and those who did not (20 vs 17 months, P = 0.2488), although surgery did provide significant symptomatic and haemodynamic improvements, as well as some with long-term survival. A trend towards better OS was demonstrated for those who received both post-operative chemo- and radio-therapy compared to those who did not, with OS of 24 and 8 months, respectively (P = 0.3417).

### Exceptional survivors

The longest surviving patient (102 months) is a 70-year-old woman who presented with dyspnoea (NYHA class III) and dependent oedema. She underwent PEA for a saddle-type intimal sarcoma (R1 resection) and had no radiological evidence of metastasis. Given the high probability of cancer dissemination secondary to the vascular nature of the disease and surgical procedure, it was decided that she would benefit from adjuvant chemotherapy followed by radiotherapy. She was given epirubicin and a radical dose of radiotherapy (60 Gy in 30 fractions). She did not show any evidence of disease on follow-up CT scans until 7 years later, when she developed lung metastases. These were treated with radiofrequency ablation. Although the disease in her lungs has progressed since then, these remain of small volume and she is currently being kept under observation.

The second longest surviving patient (85 months) is a 57-year-old woman who presented with chest pain and breathlessness (NYHA class II). CT scan showed a large filling defect (maximum diameter of 4 cm) within the right main pulmonary artery that extended into the lower lobe vessels. There was no significant lymphadenopathy. She underwent PEA and histological examination confirmed intimal sarcoma with involvement of the resection margin. However, after discussion she decided not to have post-operative chemo- or radio-therapy. She remains free of disease on follow-up CT scans more than 7 years after her surgery.

## Discussion and literature review

PAS is usually asymptomatic until vessel occlusion occurs, and as such patients normally present with signs and symptoms of advanced disease. These are often suggestive of pulmonary hypertension, heart failure or thromboembolic disease. Dyspnoea is a universal complaint, although chest pain, oedema and constitutional symptoms are also frequently reported. The radiological findings can mimic pulmonary hypertension and in many cases, inappropriate thrombolytic or anti-coagulant therapy had already been given before a diagnosis of PAS was considered. Certain features on CT pulmonary angiography could help to differentiate between these two conditions [13,14]. The role of ^18^F-fluorodeoxyglucose positron emission tomography in the diagnosis of PAS is still under investigation [15,16].

The prognosis for patients with PAS is generally poor, with a median OS of approximately 17 months as reported here and by others [2,3,8]. Due to the presence of pulmonary arterial occlusion and the acute symptoms associated with this, surgical resection is usually the mainstay of therapy. Surgery for PAS can include pneumonectomy, lobectomy, PEA or tumour debulking with or without pulmonary artery reconstruction, and the choice of procedure is dependent on factors such as the tumour location and distal extension. Immediate post-operative mortality rate has been previously reported to be around 13-15%, and surgical resection was nearly never R0 [3,5]. An analysis of combined series by Blackmon *et al*. suggested that patients who underwent an attempt at curative resection have longer OS compared to those who had incomplete resection (median OS of 36.5 vs 11 months) [2]. Since PAS arises from the vascular endothelium and is often bilateral, the aim of PEA is to remove the tumour to restore blood flow to affected regions of the lungs and to relieve the associated pulmonary hypertension. For this reason, PEA often results in significant symptom improvement although the resection margins are rarely clear and therefore the procedure is deemed non-curative. However, in the unusual event of a unilateral disease that was diagnosed early, surgical cure by pneumonectomy is a possibility provided that the patient has enough functional reserve in the remaining lung. Unfortunately this was not the case for any of our patients in the series. Even though PEA did not result in a clear survival benefit, it did provide a significant alleviation of symptoms (from NYHA class III/IV to I/II). Three of our 14 patients who underwent PEA died peri-operatively, although two of them were high-risk surgical candidates following an emergency transfer from other hospitals due to rapid cardio-respiratory deterioration.

All of the notable case series published to date have focused primarily on the surgical aspect of PAS management [3,9,10], and the role of post-operative chemo- and radio-therapy remains unclear. In the combined series by Blackmon *et al*., the median survival of those who had received multimodality treatment was found to be superior to those who only had single-modality therapy (median survival of 24.7 and 8.0 months, respectively), although the latter was defined as either surgery, chemotherapy or radiotherapy alone, rather than surgery with no post-operative treatment [2]. In 2013, Mussot *et al*. reported a relatively large surgical series of 31 patients with PAS [3]. Of these, six received neoadjuvant chemotherapy. For adjuvant treatment, 15 received chemotherapy, two had radiotherapy and one patient received both. The authors concluded that there appeared to be no statistical survival benefit in those who received adjuvant treatment compared to those who did not.

A number of peri-operative chemotherapy agents have been reported in the literature for PAS, including anthracyclines, ifosfamide, gemcitabine, taxanes, platinums and immunotherapy [2,17-20]. Anthracyclines, either alone or in combination are the most commonly-used agents, as with most soft tissue sarcomas. Chemotherapy is normally given post-operatively, although cases of improved outcome with neoadjuvant chemotherapy have been described – Linden *et al*. reported a patient whereby tumour downstaging with neoadjuvant doxorubicin and ifosfamide allowed a radical pneumonectomy to be performed, resulting in a 7-year disease-free survival [7,19]. In our series, a total of four patients received trimodality treatment with post-operative chemotherapy and radiotherapy. The median OS for this group of patients was 24 months compared to 8 months for those who only had surgical resection (n = 6) or post-operative chemotherapy alone (n = 1), although this was not statistically significant. All but one patient had received an anthracycline, and they tolerated it well without evidence of cardiotoxicity despite symptoms of pulmonary hypertension before PEA. For patients with inoperable, metastatic or recurrent PAS, chemotherapy with an anthracycline or its combinations are commonly administered, but others such as gemcitabine and docetaxel, cisplatin and vinorelbine, ifosfamide, as well as radiotherapy could also provide reasonable disease control [17,21-24]. In our series, PLDH, VID, cisplatin and topotecan appeared to have some activity in PAS, although disease progression occurred within months from treatment completion.

Other than chemo- and radio-therapy, a number of reports have found that repeated surgical interventions could also be associated with prolonged survival in patients with metastatic PAS [18,25,26]. This is not entirely surprising, given that surgery for limited metastatic sarcoma is well established. Indeed, we were able to achieve some degree of disease control with radiofrequency ablation for our longest surviving patient who developed lung metastases 7 years after her initial diagnosis.

An inherent shortcoming with a study of such a rare disease is the small sample size. Another limitation of our series is the lack of complete data for some patients that were discharged back to their local hospitals. We do not have information on any post-operative treatment of one patient, as well as details of palliative chemo- and/or radio-therapy of another five patients, although these were censored accordingly on their last follow-up visits in the survival analysis.

Is there a role for targeted therapy in PAS? The only targeted agent approved for use in soft tissue sarcoma at present is the tyrosine kinase inhibitor pazopanib, based on the results of the PALETTE trial [27]. Its use in PAS has not been reported – this is likely due to the rarity and aggressiveness of the disease, and its associated risk of bleeding and thrombosis that could be viewed as relative contra-indications for treatment with pazopanib. A frequent observation in the molecular analysis of intimal sarcoma is gains and amplifications in the chromosomal region of 12q13-14 and the overexpression of Mdm2, a negative regulator of p53 [28,29]. Furthermore, amplification of genes encoding for the platelet-derived growth factor receptor α (*PDGFA*), epidermal growth factor receptor (*EGFR*), cyclin-dependent kinase 4 (*CDK4*) and Gli1 (*GLI1*) are also commonly found [29-31]. Targeting these aberrations with small molecule inhibitors might someday prove beneficial in controlling this devastating disease.

## Conclusions

Patients with PAS often have acute symptoms mimicking pulmonary hypertension, heart failure or thromboembolic disease. A complete surgical cure is rarely possible as it usually presents late as bilateral, locally-advanced and/or metastatic disease. As such PAS carries a dire prognosis, with a median OS of only 17 months. In our experience, PEA provides good symptomatic relief to patients and in some cases offers a chance of long-term survival. Adjuvant chemo- and radio-therapy may confer a survival benefit but further studies are needed to confirm this. For patients with inoperable and/or metastatic PAS, palliative chemo- and radio-therapy offer a reasonable chance of disease control and should be considered in all cases.
